# *In Vivo* Circadian Oscillation of dCREB2 and NF-κB Activity in the *Drosophila* Nervous System

**DOI:** 10.1371/journal.pone.0045130

**Published:** 2012-10-15

**Authors:** Anne K. Tanenhaus, Jiabin Zhang, Jerry C. P. Yin

**Affiliations:** 1 Neuroscience Training Program, University of Wisconsin-Madison, Madison, WI 53706; 2 Departments of Genetics and Neurology, University of Wisconsin-Madison, Madison, WI 53706; Washington University Medical School, United States of America

## Abstract

cAMP response element-binding protein (CREB) and nuclear factor kappa-B (NF-κB) are two ubiquitous transcription factors involved in a wide number of cellular processes, including the circadian system. Many previous studies on these factors use cellular assays that provide limited information on circadian activity or anatomical specificity. The ability to study transcription factors in defined tissue within intact animals will help to bridge the gap between cellular and *in vivo* data. We have used the GAL4-UAS and FLP-FRT systems to gain spatial control over reporter gene expression. Using a luciferase-based reporter, we show *in vivo* that *Drosophila* dCREB2- and NF-κB-mediated transcription oscillates in neuronal cells, glia, and in the mushroom body, a higher-order brain center in flies. This oscillation is under circadian control, cycling with a 24-hour rhythm, under both light-dark and dark-dark conditions. In light-light conditions, dCREB2 and NF-κB reporter flies exhibit a suppression of rhythmic activity. Furthermore, neuronal cycling of dCREB2 and NF-κB activity are modulated in *period* mutant flies, indicating these oscillations are controlled through the central clock. This study shows for the first time region-specific circadian oscillation of dCREB2/NF-κB activity in the *Drosophila* nervous system.

## Introduction

The biological importance of circadian rhythms is firmly established and widely accepted. The daily oscillation of internal function and behavioral output are present throughout evolution, and are conserved from invertebrates to humans. This circadian activity is self-sustaining, and can be synchronized to the local environment. Disruption of these rhythms has detrimental effects on health and cognitive function [1], [2].

Cell-autonomous molecular oscillators govern circadian rhythms. In flies, the central pacemaker consists of a network of molecular interactions in a small population of cells. At the core of this machinery is a transcriptional feedback loop: the heterodimer CLOCK/CYCLE (CLK/CYC) activate the transcription of *period* (*per*) and *timeless* (*tim*). Once accumulation of cytoplasmic PER and TIM proteins is sufficient, they enter the nucleus and inhibit CLK/CYC, inhibiting transcriptional activity. The presence of these molecular oscillators in a subset of clock cells is sufficient to drive rhythmic responses throughout the organism [3].

Circadian changes in the expression of a large number of genes have been identified across many tissues in *Drosophila* [4]–[11]. While all of these studies identify transcripts with circadian expression patterns, there is often little overlap in the specific genes that are identified [12]. Identifying transcription factors that are under circadian regulation could provide a means of simplifying the interpretation of this data. However, whether most transcription factors have oscillations in their activities is unknown. In this study, we focus on two ubiquitous transcription factor families that have been linked to the circadian system: cAMP response element-binding protein (CREB) and nuclear factor kappa-B (NF-κB).

The activity of the *Drosophila* CREB homolog (dCREB2) is under circadian regulation, oscillating in a 24-hour rhythm with nighttime and daytime peaks in activity [13]. Circadian control of CREB activity has important physiological implications. For example, CREB mediates the circadian modulation of gluconeogenesis in mice [14]. There is also evidence that CREB acts upstream of the circadian system. In the suprachiasmatic nucleus (SCN), the master circadian pacemaker in mammals, CREB plays a role in photic entrainment [15]–[19]. Suppression of CREB in the SCN inhibits expression of PER1 and PER2 [19]. Likewise, a mutation in dCREB2 shortens the 24-hour circadian rhythm in flies, and impairs the oscillation of *per* [13]. Recently, cAMP signaling has been demonstrated as an integral component of rhythmicity in the SCN [20]–[22].

There are similar indications that NF-κB interacts with the circadian system in mammals. NF-κB is present in the hamster SCN, and inhibition of NF-κB blocks light-induced phase shifts [23]. Circadian variation in NF-κB activity has also been detected in the rat pineal gland [24]. Finally, effects of the circadian clock on apoptosis are mediated by NF-κB activity in mouse cancer models [25]. In *Drosophila*, the effects of the immune response on sleep are under circadian control, and may require the NF-κB family member *Relish* [26].

Luciferase-based transcription factor reporters have provided significant insight into the function and regulation of the molecular clock [27]–[33]. Because luciferase activity is short-lived [34], and can be measured in behaving animals over extended periods of time, these reporters are powerful tools for measuring changes in molecular activities *in vivo*. Despite the advantages of this approach, it has rarely been applied outside of the core molecular clock to measure the general activity of transcription factors over the day∶night cycle. We previously used a luciferase-based reporter gene under the control of dCREB2 binding sites (CRE-luc) to show that dCREB2 activity cycles [13]. An earlier report of NF-κB activity in flies utilized a reporter similar to the CRE-luc reporter, but it was not measured across the day/night cycle [26]. Until now, the major disadvantage of this approach has been the inability to refine these measurements to specific tissues *in vivo*.

To gain spatial control of CRE-luc reporter activity, we take advantage of the UAS-GAL4 [35] and FLP-FRT [36] systems to restrict luciferase expression to specific populations of cells. We use the same strategy to generate a reporter fly based on a consensus sequence for NF-κB. In this study, we provide evidence *in vivo* that both dCREB2 and NF-κB transcriptional activities oscillate in multiple subsets of cells in the *Drosophila* nervous system. We show, furthermore, that these oscillations are under circadian control, and are modulated in *per* mutant flies.

## Results

**Figure 1:**
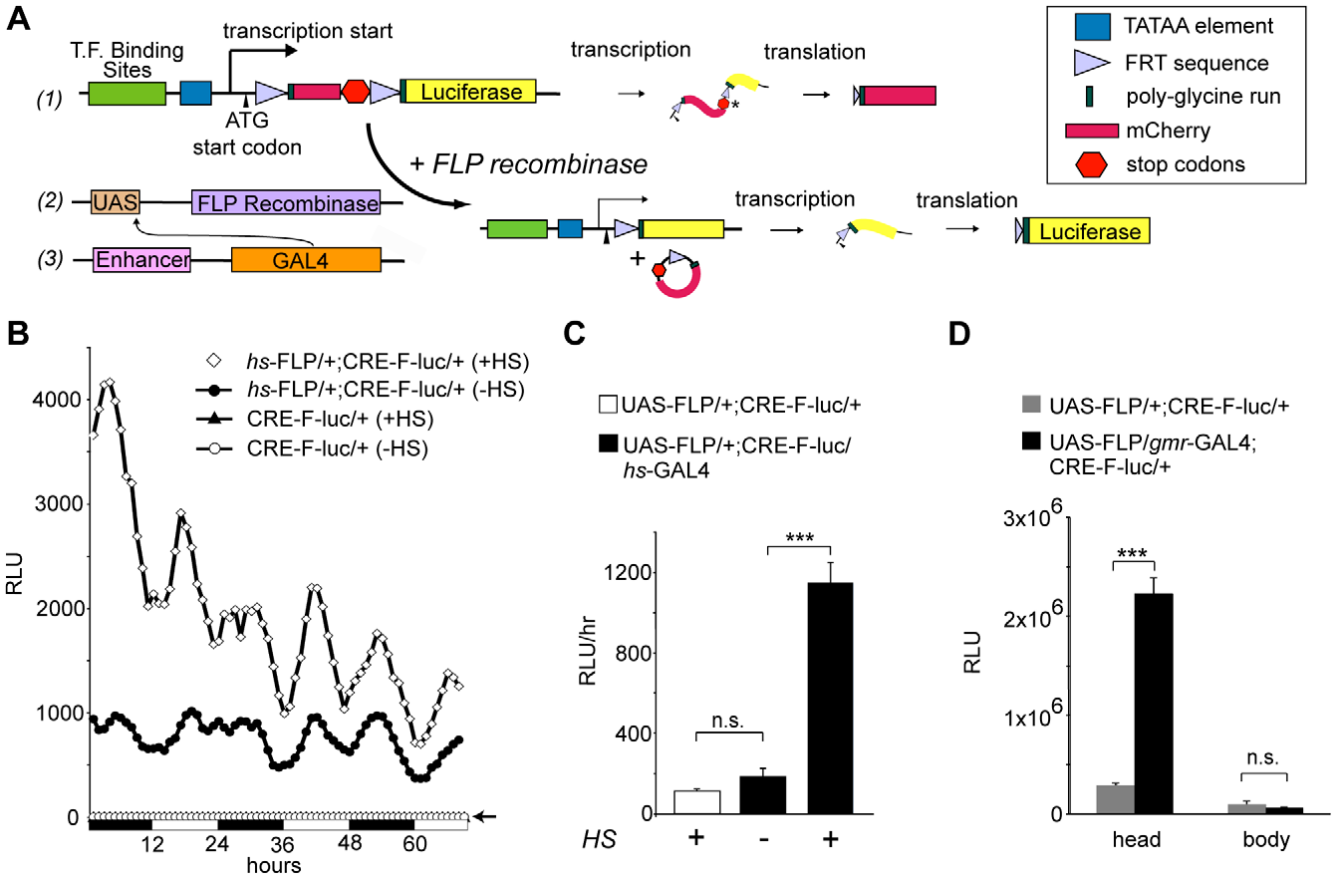
Developing a spatially restricted reporter system. (A) A cartoon of the reporter system. Three DNA binding sites are placed upstream of the CaSpeR TATAA sequence, followed by transcription (arrow) and translation (arrowhead) initiation sites, an FRT-flanked open reading frame for mCherry (ORF) including two tandem stop codons, and the luciferase ORF (#1). Targeted GAL4 expression (#3) drives the expression of UAS-FLP (#2), which catalyzes site-specific recombination at the FRT sites, activating the reporter. (B) CRE-F-luc reporter activity is FLP-dependent. Singly (CRE-F-luc) or doubly transgeneic flies (*hs*-FLP/+;CRE-F-luc/+) are maintained under 12∶12 LD conditions. Flies are exposed to heat-shock (+HS) or not (−HS), and measured for *in vivo* luminescence. The relative luminescence is plotted as a function of time, with daytime (white bars) and nighttime (black bars) durations indicated below the graph. Each data point represents the average hourly luminescence counts in relative light units (RLU) of 24 flies. (C) FLP protein expressed using the GAL4-UAS system can activate the reporter. Reporter activity (Y-axis, the mean hourly relative light units [RLUs] over a 4-day window) is plotted as a function of genotype (indicated with different colored bars) and treatment (+/− HS, heat shock). Error bars = S.E.M. *** p<0.001, Student' T-test, n = 24; n.s. signifies not significant. Similar statistical comparisons are made for the remaining figures. (D) Anatomical specificity of *gmr^long^*-GAL4 driven reporters. *In vitro* luciferase activity measured in extracts made from isolated heads and bodies. The relative light units (Y-axis) are plotted as a function of the genotype (shown in gray [UAS-FLP/+; CRE-F-luc/+ or black [UAS-FLP/*gmr^long^*-GAL4; CRE-F-luc/+]) or tissue source (head versus body).

We generated modified versions of the CRE-luc reporter [13] to measure transcriptional activity *in vivo* in defined subsets of cells. A reporter transgene was placed under the control of the FLP-FRT and GAL-UAS systems to allow for spatial control over reporter expression (**Fig. 1A**). The new reporters contain three binding sites for dCREB2 (or NF-κB) upstream of the CaSpeR TATAA element. Downstream of where transcription initiates, a Cavener sequence [37] and translation initiation codon are positioned 5′ to an FRT-flanked cassette containing the coding sequence for mCherry (but without its normal start codon). The coding region terminates with two tandem stop codons. Translation that initiates at the ATG codon reads through the first FRT site and the mCherry sequence before terminating at the tandem stop codons. The open reading frame for firefly luciferase (without its normal start codon) is placed downstream of the cassette. In the absence of FLP, translation should terminate at the tandem stop codons just 3′ to the mCherry sequence. In the presence of FLP, site-specific recombination between the FRT sites should remove most of the cassette, leaving one FRT site. Translation initiated at the first ATG codon should then read through the remaining FRT sequence, a poly-glycine encoding linker, and into the luciferase sequence, producing a fusion protein with luminescent activity. As a result, specific reporter expression should be activated when FLP recombinase is expressed under the control of an anatomically defined GAL4 driver.

To test if FLP recombinase can activate luciferase expression, we combined the CRE-F-luc transgene (**Fig. 1A #1**) with a heat-shock inducible transgene expressing FLP recombinase, and measured luciferase activity over time in live flies (**Fig. 1B**). While flies carrying the CRE-F-luc transgene alone show no detectible luminescence, flies carrying an additional *hs*-FLP transgene exhibit substantial leaky reporter expression when maintained at 22°C. This activity increases significantly when these flies are exposed to heat shock. Similar to the previous reporter [13], the CRE-F-luc reporter shows an oscillating pattern of day-night activity, characterized by daytime and nightime peaks. Light output dampens over time with luciferin degradation.

While FLP-dependence for reporter activation is important, the real utility of this system requires that GAL4-UAS mediated spatial control can be layered onto the reporter. In order to test this, we combined the CRE-F-luc transgene with a UAS-driven FLP recombinase transgene [38]. Baseline expression in live UAS-FLP/+;CRE-F-luc/+ flies is detectable but low (∼100 RLU/hr), and does not significantly increase in *hs*-GAl4/UAS-FLP/CRE-F-luc flies in the absence of heat shock (**Fig. 1C**). However, luciferase activity increases significantly in these triply transgenic flies following heat shock. Therefore, FLP recombinase expression under UAS control activates the reporter.

To verify the specificity of the fully assembled reporter, UAS-FLP;CRE-F-luc flies were crossed to an eye specific driver line, *gmr^long^*-GAL4. Luciferase activity was then measured from isolated fly heads. This activity shows a substantial *gmr^long^*-driver-dependent increase. However, there is no significant difference between groups when isolated bodies are measured (**Fig. 1D**), indicating specific reporter expression. Measurements of dissected head and eye tissue confirm that the signal is produced only in the eye tissue (**Fig. S1; see Methods S1 for details**).

In order to characterize the range of signal detection, and to assess the contribution of various cell populations to circadian oscillations in dCREB2 activity, we screened a number of GAL4 driver lines for reporter activity. Flies containing both the UAS-FLP and CRE-F-luc transgenes were crossed to different GAL4 driver lines, or to wild type flies as a baseline control. In most of the lines tested, significant luciferase activity is detected above baseline (**Table S1**). A significant increase in reporter activity above baseline is detectible when GAL4 is driven in small populations of neurons in the ellipsoid body (*c42*-GAL4 or *c232*-GAL4) or in the mushroom body (MB, *ok107*-GAL4) (**Table S1,** **Fig. 3C** **)**, indicating that the reporter can be used to measure activity in small subsets of cells. However, no signal is detectable using the *npf*-GAL4 driver (**Table S1**), which expresses in only a handful of cells [39]. Notably, all drivers that produce detectible activity exhibit oscillating activity with the same general temporal pattern, peaking at around zeitgeber time (ZT) 4 (mid-day) and ZT16 (mid-evening).

**Figure 2:**
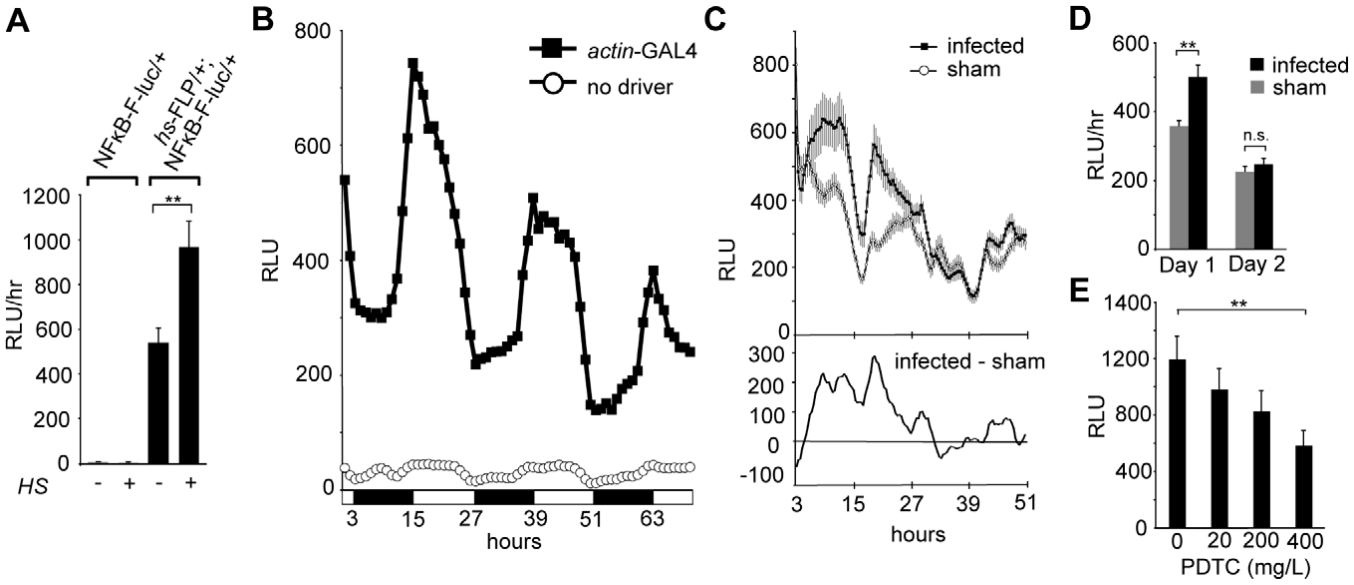
Expression of the NF-κB-F-luc reporter is FLP dependent, oscillatory and NF-κB-dependent. (A) NF-κB-F-luc reporter activity is FLP-dependent. Reporter activity (Y-axis, relative light units) is plotted as a function of genotype (singly [NFκB-F-luc] or doubly transgenic flies [hs-FLP/+;NFκB-F-luc/+] and treatment (heat shock HS+ or not, HS−). The histogram bars show the mean hourly counts over a 3-day window (n = 24). (B) The ubiquitously expressed *actin*-GAL4 driver produces oscillatory NFκB-F-luc activity over a 24 h period. Luciferase activity is plotted as a function of time for singly (denoted no driver; NF-κB-F-luc) or doubly transgenic (denoted *actin*-GAL4; NFκB-F-luc/*actin*-GAL4) flies. n = 24 for each group (C) Bacterial infection affects reporter activity. Top panel: Reporter activity in the fat body is plotted for the first 48 hours after infection (filled circles) or sham treatment (open circles). The *cg*-GAL4 line (together with UAS-FLP and NFκB-F-luc) is used to activate the reporter in the fat body. n = 48 for each group. Bottom panel: The difference in reporter activity between the infected and sham-treated groups (infected RLU-sham RLU) over the same duration post-injection. (D) Quantification of reporter activity (mean hourly count) during the first and second days following infection. The mean luminescence counts in RLU are plotted as a function of time after infection. Values for each day are averaged and binned together. (E) There is a dose-dependent decrease in reporter activity in flies fed PDTC. The relative luminescence is plotted as a function of the PDTC dose fed to flies. Triply transgenic flies (UAS-FLP/+;NF-κB-F-luc/*actin*-GAL4) were fed different dosages of PDTC for 24 hours and then measured for luminescence 1 h after the end of feeding. n = 24 for each group, Error bars = S.E.M, **p<.001.

We used the same strategy to build a reporter for general NF-κB activity. Our reporter is similar to a previous NF-κB luciferase reporter, except that it is FLP-dependent, and based on a more generalized NF-κB binding site [26]. As with the CRE-F-luc reporter, luciferase activity is undetectable in flies that contain only the NFκB-F-luc reporter, but FLP expression stimulates activity (**Fig. 2A**). When ubiquitously expressed under the control of an *actin*-GAL4 driver, NF-κB reporter activity oscillates in a 24 hr rhythm under a light∶dark regimen, peaking shortly after lights on (ZT1) (**Fig. 2B**). We observe a similar pattern when the reporter is activated ubiquitously by *hs*-FLP (**Fig. S2**).

To verify that the NF-κB-F-luc reporter responds to NF-κB activity, we asked if established modulators of NF-κB would affect reporter activity. Because the innate immune response activates NF-κB [40], [26], bacterial infection should stimulate activity measured from NFκB-F-luc reporter flies. As the fat body is the center for innate immunity in *Drosophila*, we used a fat body-specific driver (*cg*-GAL4) to express the reporter in this tissue. These flies were inoculated with either a mixture of gram-negative and gram-positive bacteria, or received a sham injury. Bacterial infection, when compared to mock injection, significantly increases NFκB-F-luc activity (**Fig. 2C,D**). This response peaks between 6 and 20 hours following infection, and lasts for about 30 hours.

To validate the specificity of the reporter, we asked if inhibiting NF-κB activity would decrease NFκB-F-luc activity. Pyrrolidine dithiocarbamate (PDTC) is a potent NF-κB inhibitor, suppressing NF-κB signaling via stabilization of I-κBα and inhibition of the ubiquitin-proteasome pathway [41]–[46]. Feeding of this drug has been used in *Drosophila* to suppress NF-κB activity [47]. Female UAS-FLP/+;NFκB-F-luc/*actin*-GAL4 flies were maintained for 24 h on yeast paste containing PDTC and luciferin, and luciferase activity was measured 1 h later *in vivo*. Reporter activity is reduced in a dose-dependent manner (**Fig. 2E**). At the highest concentration (400 mg/L), significant suppression of reporter activity lasts for 24 h (**Fig. S3**).

**Figure 3:**
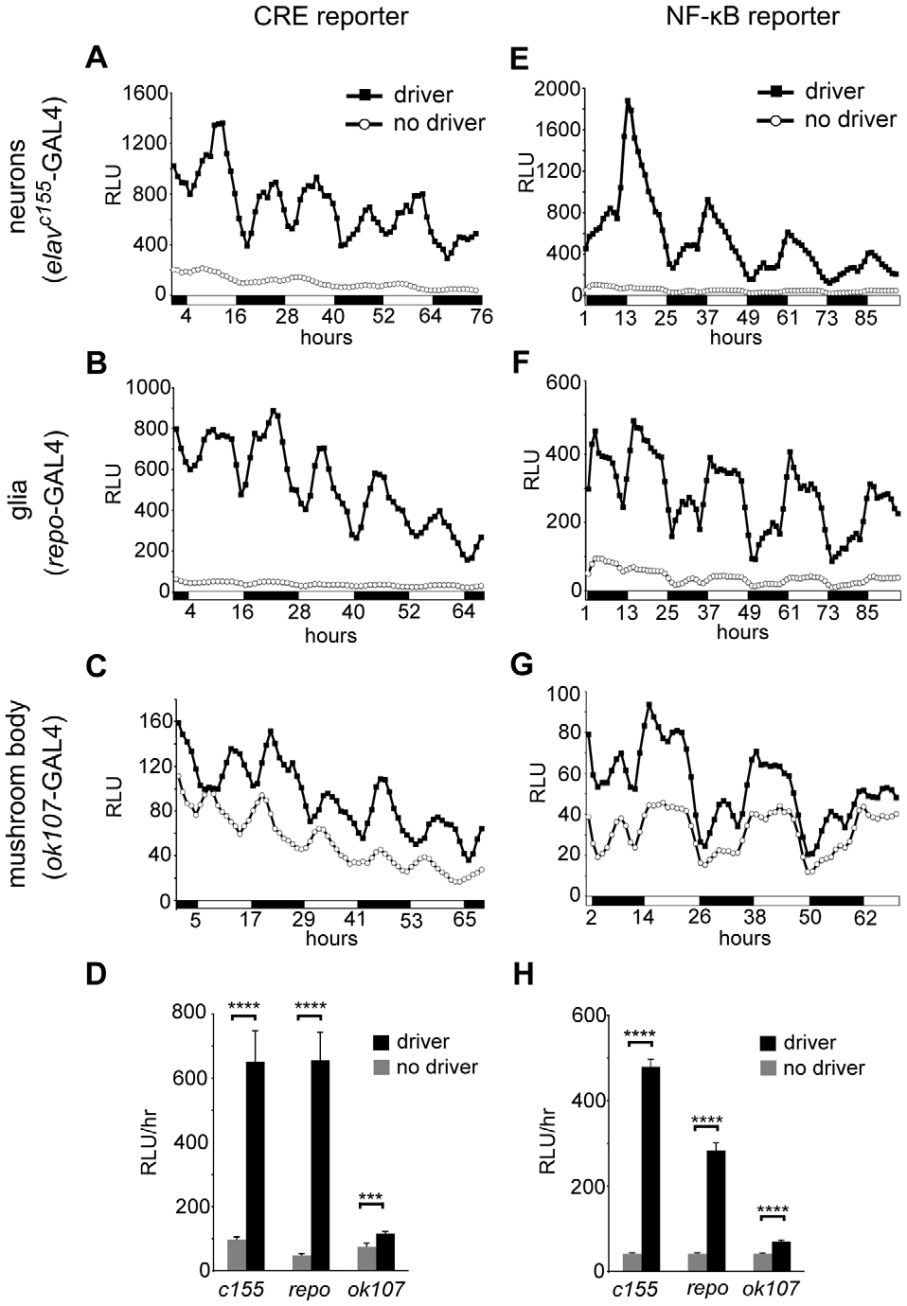
dCREB2 and NF-κB activity cycle in multiple tissues. Reporter activity is plotted as a function of circadian time. Each different cellular population is described on the left, and the tissue-specific driver used to activate the reporter is shown. In each panel, a comparison is shown between the background (denoted no driver) and activated (driver) expression levels. The activity is shown for the CRE-F-luc (panels A–C) and the NFκB-F-luc (panels E–G) reporters in neurons (panels A and E), glia (panels B and F) and in the mushroom body (panels C and G). The average activity over the duration of the experiment for each driver is quantified for the CRE-F-luc (panel D) and NFκB-F-luc (panel H) reporters. All bars represent mean hourly counts from 24–28 flies. Error bars = S.E.M, ***p<0.001, **** p<0.0001.

To measure the temporal pattern of dCREB2 and NF-κB activity in the nervous system, we activated the reporter under the control of the pan-neuronal *elav* ^c155^-GAL4 (**Fig. 3A, E**), the pan-glial *repo*-Gal4 (**Fig. 3B,F**), or the MB-specific *ok107*-GAL4 drivers (**Fig. 3C,G**). For both reporters, we observe significant signal with each of the three drivers when compared to flies carrying the reporter and UAS-FLP transgenes alone (**Fig. 3D,H**). CRE-F-luc activity in neurons, glia, and the MB shows a similar diurnal pattern, with broad peaks during the middle of the day and the middle of the night (**Fig. 3A–C**). NF-κB reporter activity shows a similar oscillating pattern in neurons, glia, and the MB, with a major peak shortly after lights on (**Fig. 3E–G**). Interestingly, unlike with the *actin*-GAL4 driver (**Fig. 2B**), a secondary peak in NF-κB reporter activity in the middle of the nighttime period is also present in neurons, glia, and the MB (**Fig. 3E–G**). In general, there is some variability in the presence of the secondary peak (data not shown), consistent with mixed reports of secondary peaks in *per* reporter activity [27], [28], [48], [49]. This issue requires further investigation.

**Figure 4:**
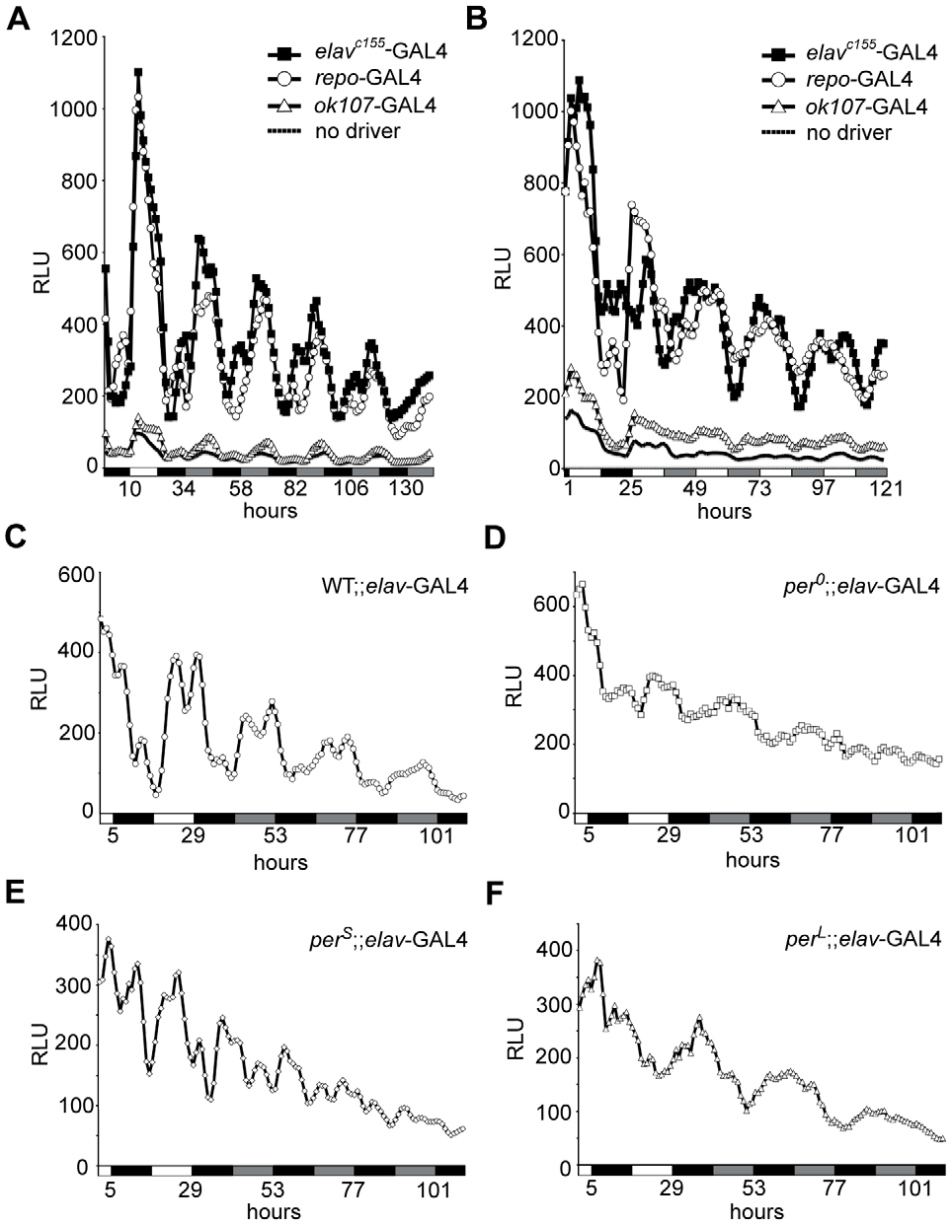
The NF-κB reporter activity oscillates under circadian control. (A) NF-κB reporter activity in multiple tissues oscillates in constant darkness. Reporter activity is plotted as a function of time for different genotypes. Triply transgenic flies, where the reporter is activated in all neurons (using the *elav* ^c155^-GAL4 driver, black squares), in a pan-glial pattern (using the *repo*-GAL4 driver, open circles), or in the mushroom body (using the *ok107*-GAL4 driver, open triangles), exhibit oscillatory activity when flies are maintained in light∶dark (first 24 h period) or dark∶dark (subsequent time) conditions. These plots are compared to those from doubly transgenic flies (solid line; UAS-FLP/+; NFκB-F-luc/+) that do not contain the tissue-specific driver. (B) The activity of the NF-κB reporter in different tissues is dampened under constant light. Reporter activity is shown as a function of time, with the shift to constant light occurring during the second 24 h period. The reporter is activated in triply transgenic flies in neurons (denoted *elav^c155^*-GAL4, *elav^c155^*-GAL4; UAS-FLP/+; NFκB-F-luc/+), glia (denoted *repo*-GAL4, UAS-FLP; NFκB-F-luc/*repo*-GAL4) or the mushroom body (denoted *ok107-GAL4*; UAS-FLP; NFκB-F-luc/+; *ok107*-GAL4/+) when compared to doubly transgenic flies without a GAL4 driver (solid line, UAS-FLP/+; NFκB-F-luc/+). (C–F) Neuronal reporter activity is plotted over time as flies are shifted from light∶dark to constant darkness. For all of these panels, the same transgenes (UAS-FLP/+; NFκB-F-luc/*elav*-GAL4) exist in all flies, but the flies are examined in a wild type (C), *per^0^* (D), *per^S^* (E) or *per^L^* (F) genetic background.

Because NF-κB reporter activity shows a robust rhythmic activity, we asked whether the reporter oscillates under circadian control. Robust oscillations in neurons, glia, and the MB are maintained in constant darkness after light∶dark entrainment (**Fig. 4A**). Under LL conditions (see Methods), oscillations are maintained, but dampen over the course of light exposure (**Fig. 4B**). The persistence of oscillating activity in light-entrained flies maintained in constant darkness is strong behavioral evidence for circadian control of reporter activity. To determine if this activity is genetically under circadian control, we asked if mutations in the *period* gene (*per^0^*, *per^S^* and *per^L^*) affect reporter activity. Flies were raised under a 12∶12 light∶dark cycle, and switched into constant darkness. As previously observed, neuronal NF-κB reporter activity is maintained under DD conditions in wild type flies (**Fig. 4C**). In contrast, NF-κB reporter activity in *per^0^* flies becomes arrhythmic (**Fig. 3D**), activity in *per^S^* mutant flies shows a shorter period (∼19 h, **Fig. 3E**), while *per^L^* flies have an extended free-running period (∼28, **Fig. 3F**). In the original CRE-luc reporter, these mutations in *per* affected dCREB2 reporter activity in an almost identical manner [13]. dCREB2 reporter activity in neurons is similarly modulated in *per^0^*, *per^S^,* and *per^L^* mutants (**Fig. S4**). Collectively, this data suggest that dCREB2 and NF-κB binding activity are likely to be under circadian control in most, if not all, nervous system tissues.

**Figure 5:**
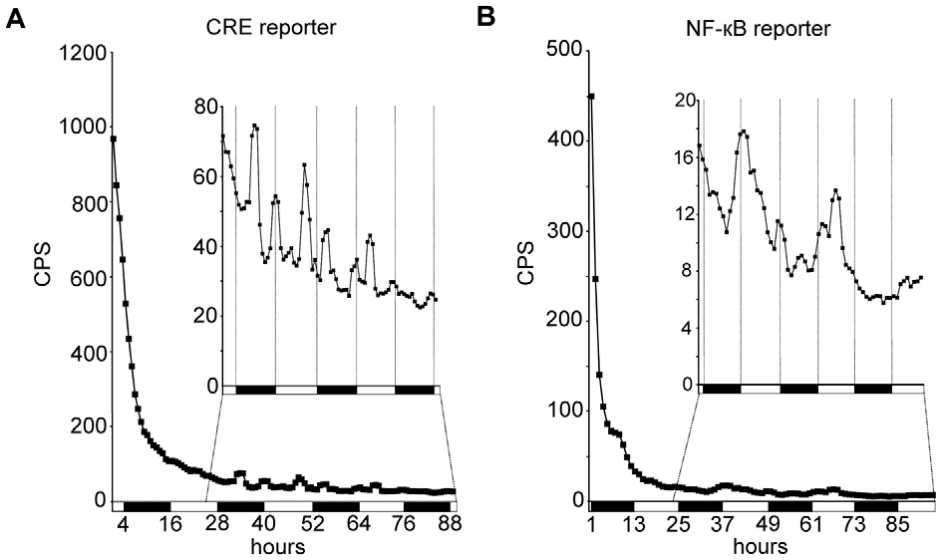
Oscillations in dCREB2 and NF-κB activity persist after luciferin removal. (A) Reporter activity in UAS-FLP/+;CRE-F-luc/*actin*-GAL4 flies starting ∼1 h following luciferin removal, plotted as a function of time. The inset shows a scaled view of the same data, with the first 24 h dropped. (B) Reporter activity in UAS-FLP/+;NFκB-F-luc/*actin*-GAL4 flies starting ∼1 h following luciferin removal. Inset shows a scaled view of the same data, with the first 24 h dropped. n = 24 flies for each group.

Because feeding behavior is also under circadian regulation [50], circadian changes in luminescence in dCREB2 and NF-κB reporter flies might be explained by diurnal variations in substrate availability due to feeding. To test this, we asked if oscillations in reporter activity persist after flies are removed from luciferin. UAS-FLP/+;CRE-F-luc/*actin*-GAL4 or UAS-FLP/+;NFκB-F-luc/*actin*-GAL4 reporter flies were kept on luciferin-containing food for 24 hours, and then transferred to 96-well plates containing non-luciferin food. Luminescence decayed rapidly, with a half-life of around 3.5–4 hours for the dCREB2 reporter flies, and 2.5 hours for the NF-κB reporter flies (**Fig. 5**). However, oscillations in residual activity from both reporters are detectable for multiple days after substrate removal (**Fig. 5** insets). A similar pattern of activity occurs for UAS-FLP/+;CRE-F-luc/*actin*-GAL4 flies whether maintained on, or removed from luciferin-containing food, with largely two peaks during the daytime and the nighttime (Fig. 1B, 3, 5A). Daytime and nighttime peaks of luciferase activity also persist beyond 24 h after substrate removal in CRE-luc reporter flies (**Fig. S5**). Likewise, UAS-FLP/+;NFκB-F-luc/*actin*-GAL4 reporter flies show the same general activity pattern both on and off of luciferin, with one dominant peak occurring shortly after lights-on (**Fig. 2B** **,** **5**). Upon substrate removal, reporter activity patterns become more variable, perhaps attributable to increased variability of signal when luciferin is low, greater variability in residual substrate between flies, or because substrate ingestion has some contribution to reporter activity patterns. Regardless, circadian variation in substrate feeding does not itself appear to explain circadian variation in reporter activity.

## Discussion

We have used the FLP/FRT system to make spatially restricted transcription factor reporters. These transgenes contain reiterated consensus DNA binding sequences for single factors, thus providing a simpler, but cleaner picture of the activation state of specific transcription factors. The use of luciferase as the reporter moiety, which shortens the half-life of reporter activity, allows a more dynamic picture of transcription. This new reporter format produces detectable signal from behaving flies that is FLP-dependent (**Fig. 1B–C** **,** **2A** **, S1**). This allows the activity of specific factors to be measured in defined cell populations.

In general, the new CRE-F-luc reporter seems to be indistinguishable from the original CRE-luc reporter [13]. Both reporters show identical temporal patterns of activity, and *per* mutations modulate them indistinguishably (**Fig. 1B** **,** **3A–D** **, S4**). When the CRE-F-luc reporter is expressed in certain small clusters of cells, activity cannot be measured, precluding us from concluding that dCREB2 activity cycles everywhere in the adult head **(Table S1**). However, since most major cell types and anatomical structures show oscillatory activity (**Fig. 3A–D** **,** **Table S1**), we suspect that dCREB2's activity oscillates ubiquitously. This suggests that the experimentally observed oscillating activity does not result from the activity of certain cells dominating the measured signal. Instead, the general diurnal pattern seems to occur when the reporter is activated in most, if not all, cells.

The NF-κB transcription factor reporter also oscillates, and is directly or indirectly under the control of the circadian system (**Fig. 2B** **,** **3E–H** **,** **4**). The *Drosophila* genome encodes three NF-κB family members, characterized by a conserved DNA binding domain: Dorsal, DIF (dorsal-related immunity factor) and Relish. These proteins can form homodimers or heterodimers to regulate transcription. Because NFκB-F-luc reporter activity responds to known modulators of NF-κB activity (**Fig. 2C–E**), the oscillations we observe seem to reflect genuine changes in NF-κB transcriptional activity across the daytime and nighttime. However, we cannot discriminate which of the forms of NF-κB controls expression off of our reporter since Dorsal, Relish and Dif/Relish can bind to the consensus binding site used in our reporter fly [51].

Low-amplitude circadian fluctuations in luciferase reporter activity, even in predicted non-circadian reporters, are common [28], though it is unclear if these rhythms are due to changes in transcription, or to non-transcriptional circadian changes that might affect luciferase activity (such as substrate feeding or locomotor activity). We can not definitively exclude the possibility that the oscillations in dCREB2 and NF-κB reporter activity are due to, or include, other non-transcriptional changes that are under circadian regulation. However, dCREB2 and NF-κB reporter oscillations do not appear to be explained by diurnal variations in substrate ingestion (**Fig. 5** **, S5**). Furthermore, the relative changes in reporter activity that we observe are stronger than the low-amplitude changes (≤0.25 fold) reported in *hsp*-luc control reporters [28], and in another transcription factor reporter that we have analyzed (data not shown). Finally, under light∶dark conditions, the dCREB2 and NF-κB reporters show different kinetics, with the onset and peak of NF-κB activity (ZT≈1) occurring during a relative trough in dCREB2 activity (**Fig. 1B** **,** **2B** **,** **3** **, S2**). It seems most likely, therefore, that dCREB2 and NF-κB reporter oscillations represent genuine changes in transcriptional activity, rather than downstream artifacts.

We detect oscillatory dCREB2 and NF-κB reporter activity in neurons, glia, and the MB (**Fig. 3**). While the specific roles of circadian activity in these particular tissues are unclear, oscillating transcription may be relevant to circadian-regulated functions in these areas. For example, a role for glial function in circadian behavior has recently emerged [52], [53]. This raises the question of whether these oscillations in glia are required for any behavioral rhythmicity or if they modulate other physiological functions. In *Drosophila*, the MB is important for sleep regulation, a process that is an output of both circadian control and homeostatic drive [54], [55]. dCREB2 has been linked to the clock [13] and has a non-circadian role in sleep-wake homeostasis [56]. In addition, manipulation of PKA activity in MB affects sleep [54]. Further work is needed to determine if dCREB2 oscillation in the MB relates to the sleep-wake cycle. The MB is also an important learning and memory center. Although the role of dCREB2 in the MB for olfactory long-term memory formation is controversial [57], a long-term molecular memory trace may require dCREB2 in MB [58]. Given recent mammalian data that suggest a role for circadian MAPK oscillations in memory maintenance [59], the possibility that dCREB2 activity cycling in the MB could be functionally important for memory processing is particularly intriguing. In mammals, NF-κB plays roles in both pathological conditions of neuronal disease [60] and activity-dependent neural plasticity [61]. The functions of NF-κB in the *Drosophila* nervous system have not been extensively studied, although previous work shows that this family of transcription factors is important in immune function. Our NF-κB reporter fly provides another tool to study its function in both pathological and physiological conditions.

Our results suggest that the overall activities of these transcription factors have relatively simple diurnal patterns of activation *in vivo*, and that this is downstream of circadian oscillators. It is also commonly understood that both of these transcription factor families respond to a variety of acute and extra-cellular stimuli in cells. There are a number of non-mutually exclusive possibilities for how the overall patterns of activity that we observe could relate to acute responses that are occurring in the organism. First, there may be some stimuli *in vivo*, downstream of circadian activity, that are especially dominant during the mid-day and nighttime periods, producing peaks. The stimuli at these particular times may be targeted to most if not all cells, thus producing a strong signal. These possibilities are especially intriguing given that much of the reporter activity we observe occurs during time periods when flies typically sleep [62], [63], a state characterized by neural synchronization in mammals [64]. Whether fly brain neurons show sleep-dependent synchronization is unknown, but there is some evidence that there are changes in neuronal activity during sleep [65]. How the patterned activity of these transcription factors relates to sleep state is an interesting question for future studies. Another possibility is that the other types of cues that can activate these transcription factors may be less global and more cell and tissue specific, thus producing limited activation. The resulting peaks in activation may be obscured beneath a background level of net circadian activity. Finally, dCREB2 and NF-κB may indeed respond to a plethora of signals, but *in vivo* these signals are synchronized by the circadian system to occur mostly during the two periods of peak activity. This last possibility implies that many physiological processes and their intercellular signals may be under circadian control in the animal, and occur at common times.

In this study, we focus primarily on the circadian pattern of dCREB2 and NF-κB activity in the nervous system. However, these factors are also involved in many other processes, including immune function [40], [66], cancer [67], aging [68], neurodegenerative diseases [69], [60] and adult neurogenesis [70]. Since thousands of *Drosophila* GAL4 lines have been characterized, this technique provides a method to non-invasively measure dCREB2 and NF-κB transcriptional activity in multiple different tissues. We believe this general approach can be used to study any transcription factor and its regulation in defined cell populations.

## Materials and Methods

### Fly Lines

The following fly stocks were used: *elav*-GAL4, UAS-FLP1, *hs*-FLP, *hs*-GAL4, *gmr^long^*-GAL4, *actin*-GAL4 (Bloomington Stock Center), *ok107-*GAL4 (kindly shared by Y. Zhong), *repo*-GAL4 (kindly shared by B. Jones), *elav^c155^*–GAL4 (kindly shared by K. Iijima-Ando), *per^0^*, *per^S^*, *per^L^* (kindly shared by A. Sehgal), and *cg*-GAL4 (kindly shared by B. Ganetzky).

### Reporter constructs

The new CRE-F-luc reporter contains three copies of the symmetrical CRE site (5′-TGACGTCA-3′) situated upstream of a canonical TATAA element. A Cavener element [37] (CAAC) and an ATG translation initiation codon are situated downstream of where transcription initiates, followed by a cassette containing FRT sites flanking the mCherry gene (without its own ATG start codon). Translation that initiates at the ATG codon would continue through the FRT site into mCherry, and would terminate at the end of the mCherry coding sequence. A short sequence coding for a poly-glycine run is downstream of the second FRT site, and placed so that it is in the same reading frame as the ATG start codon, regardless of which FRT site remains after site-specific recombination. The luciferase-coding region (minus its normal ATG start codon) is placed downstream, and in frame with, the poly-glycine run. In the absence of FLP, the transgene would produce a fusion protein with amino acids encoded by one FRT sequence, and the mCherry open reading frame. After FLP-mediated recombination, a fusion protein would be encoded that contains amino acids from the FRT sequence and a poly-glycine run, all fused to luciferase. Sequence information is available upon request.

The NFκB-F-luc reporter relies upon a κB element (5′-GGGGACTTTCC-3′) contained in the mammalian immunoglobulin/HIV promoter [71]. The design of the transgene is identical to the CRE-F-luc, except that the κB element is substituted for the CRE sites. Reporter constructs were synthesized (GenScript), sequenced, and inserted at the NotI/XhoI sites of pCaSper5. Standard methods were used to generate transgenic flies (BestGene).

### Luciferase Assays

*In vitro* biochemical assays of luciferase activity were preformed on frozen fly homogenates as previously described [72]. For the head isolation experiment, three groups of 20 flies (10 male, 10 female) were frozen and heads and bodies were isolated and homogenized in cold homogenization buffer (15 mM HEPES, 10 mM KCl, 5 mM MgCl_2_, 0.1 mM EDTA, 0.5 mM EGTA) [73]. Luciferase activity was measured in triplicate using the SteadyGlo Luciferase Assay System (Promega).

*In vivo* luciferase assays were performed as previously described [13], [27], [28]. Briefly, 24–48 flies were entrained on a 12∶12 light∶dark cycle for 4–5 days and loaded into a black 96-well microplate containing luciferin media: 1% agar 5% sucrose food containing D-Luciferin (Gold BioTechnology). 5 mM luciferin was used for most of the reporter assays: (**Fig. 1** **,** **3A–D** **, S2, Table S1**) or 25 mM luciferin was used for all others. Plates were maintained at 22°C under controlled light conditions and cycled approximately once per hour through a Packard TopCount Scintillation and Luminescence counter. For all experiments, plates were in the dark during detection periods (∼20 min/hr). The first 12–24 hours after loading are excluded from analysis to allow flies to recover from handling. A smoothing function is applied such that each data point represents the average of three measurements. For comparisons of overall activity between groups, the mean hourly reading is calculated for each fly and compared using Student's T-tests.

For pre-feeding experiments, flies were maintained in groups of 50 flies on media containing luciferin (100 mM) for 24 h before being loaded individually into 96-well microplates plates containing food alone (1% agar, 5% sucrose). The time delay between loading and the first luminescence measurement is between 30 m and 1 h.

### Drug Feeding

Pyrrolidine dithiocarbamate (PDTC, Sigma) was mixed in yeast paste containing 15 mM luciferin. Flies were maintained in standard food vials coated in yeast paste for 24 h. After drug feeding, they were transferred to 96-well plates containing standard luciferin media for 1 h before luciferase activity was measured.

### Heat Shock

For heat shock experiments, flies were maintained at 22° throughout development. Flies receiving heat shock were placed at 37° (air) for 40 minutes. Flies were allowed to recover for 24 h before all assays.

### Bacterial Infection

Micrococcus luteus (gram-positive) and Escherichia coli (gram-negative) were cultured in LB medium overnight at 37°C. A mixture of these two types of bacteria was used for bacterial infection. A thin needle was dipped in a concentrated solution of bacteria and used to prick the thorax of adult flies (10∼15-day old). Control flies received the same injury without bacteria. After infection, flies were loaded into a 96-well plate to monitor luciferase activity.

## Supporting Information

Figure S1 **Supplemental CRE-F-luc reporter validation.** (A) CRE-F-luc reporter activity is FLP-dependent. Reporter activity (Y-axis, relative light units) is plotted as a function of genotype (singly [CRE-F-luc] or doubly transgenic flies [*hs*-FLP/+;CRE-F-luc/+] and treatment (heat shock HS+ or not, HS−). The histogram bars indicate mean hourly counts over a 3-day window (n = 24). (B) Anatomical specificity of *gmr*-GAL4 driven reporter. *In vitro* luciferase activity measured in extracts made from dissected eye or remaining head tissue (n = 5). The relative light units (Y-axis) are plotted as a function of the genotype (shown in gray [UAS-FLP/+; CRE-F-luc/+ or black [UAS-FLP/*gmr^long^*-GAL4; CRE-F-luc/+]) or tissue source. (Error bars = S.E.M, **** = p<0.0001).
(TIF)

Figure S2 **NF-κB reporter activity oscillates over the day-night cycle.** FLP-activated NFκB-F-luc activity. Singly (NFκB -F-luc) or doubly transgeneic flies (*hs*-FLP/+; NFκB -F-luc/+) are maintained under 12∶12 LD conditions. Flies are exposed to heat-shock (+HS) or not (−HS), and measured for *in vivo* luminescence. The relative luminescence is plotted as a function of time, with daytime (white bars) and nighttime (black bars) conditions indicated below the graph. Each data point represents the average of 24 flies.
(TIF)

Figure S3 **PDTC inhibition of NFκB-F-luc reporter activity persists for 24 hours at high doses.** The relative luminescence is plotted with respect to the PDTC dose fed to flies. Triply transgenic flies (UAS-FLP/+;NFκB-F-luc/*actin*-GAL4) were fed different dosages of PDTC for 24 hours and then measured for luminescence 1 h after the end of feeding. Reporter activity is pooled over 1 day following 24 h PDTC feeding (n = 24 for each group) (Error bars = S.E.M, *p<.05).
(TIF)

Figure S4 **Neuronal CRE-F-luc reporter cycling is modulated in** ***per*** **mutants.** Neuronal reporter activity is plotted over time as flies are shifted from light∶dark to constant darkness. For all of these panels, the same transgenes (UAS-FLP/+; NFκB-F-luc/*elav*-GAL4) exist in all flies, but the flies are examined in a wild type (A), *per^0^* (B), *per^S^* (C) or *per^L^* (D) genetic background.
(TIF)

Figure S5 **Oscillations in CRE-luc reporter activity persist after substrate removal.** (A) Reporter activity (in relative light units, RLU) in CRE-luc reporter flies is plotted as a function of time, starting 1 h after the transfer from luciferin to non-luciferin food. Light conditions are indicated by white boxes (daytime) and grey boxes (nighttime). (B) The same data as in (A), with the first day excluded.
(TIF)

Methods S1 (DOC)

Table S1 **dCREB2 Reporter Driver Screen.**
(DOC)
