# Supplementary figures and images for: In Vivo Circadian Oscillation of dCREB2 and NF-κB Activity in the Drosophila Nervous System
Source: PLoS One. 2012 Oct 15;7(10):e45130. doi: 10.1371/journal.pone.0045130 (PMC3471920; doi:10.1371/journal.pone.0045130)

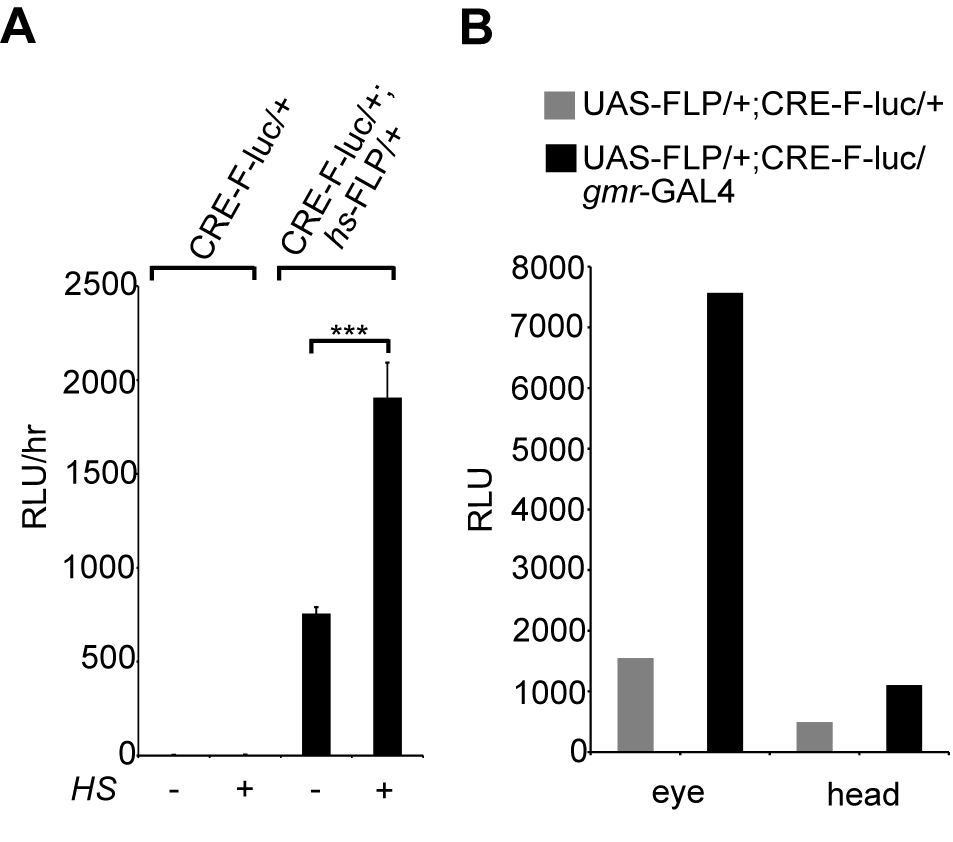

Supplement: Figure S1 — Supplemental CRE-F-luc reporter validation. (A) CRE-F-luc reporter activity is FLP-dependent. Reporter activity (Y-axis, relative light units) is plotted as a function of genotype (singly [CRE-F-luc] or doubly transgenic flies [hs-FLP/+;CRE-F-luc/+] and treatment (heat shock HS+ or not, HS−). The histogram bars indicate mean hourly counts over a 3-day window (n = 24). (B) Anatomical specificity of gmr-GAL4 driven reporter. In vitro luciferase activity measured in extracts made from dissected eye or remaining head tissue (n = 5). The relative light units (Y-axis) are plotted as a function of the genotype (shown in gray [UAS-FLP/+; CRE-F-luc/+ or black [UAS-FLP/gmrlong-GAL4; CRE-F-luc/+]) or tissue source. (Error bars = S.E.M, **** = p<0.0001). (TIF) [file pone.0045130.s001.tif]

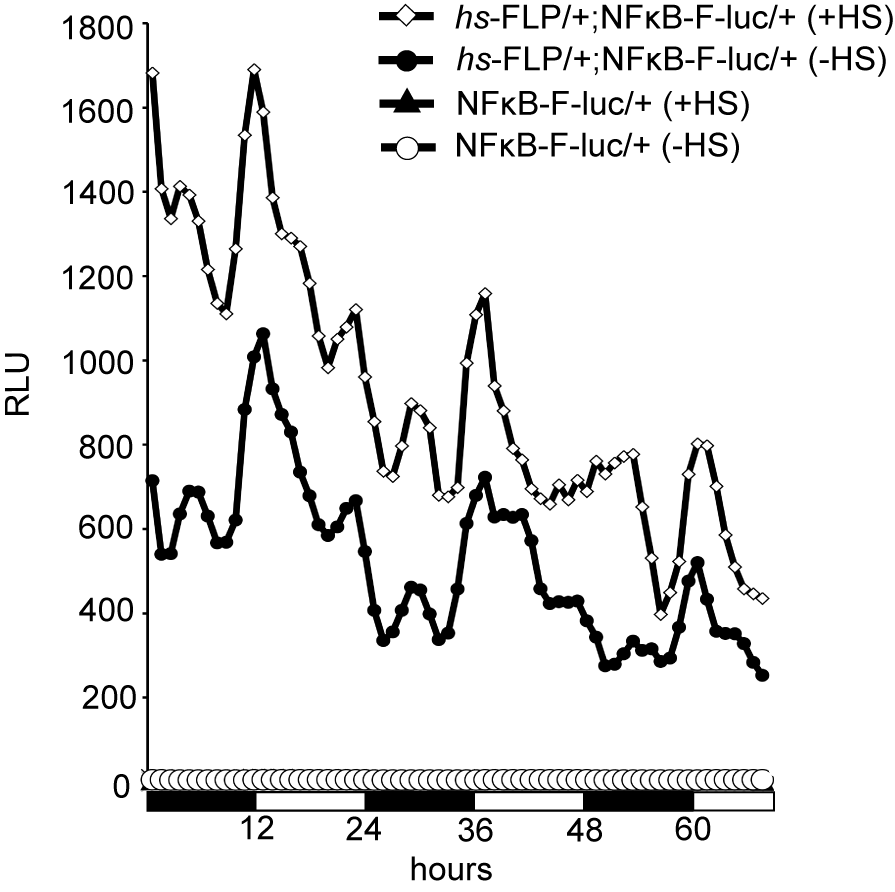

Supplement: Figure S2 — NF-κB reporter activity oscillates over the day-night cycle. FLP-activated NFκB-F-luc activity. Singly (NFκB -F-luc) or doubly transgeneic flies (hs-FLP/+; NFκB -F-luc/+) are maintained under 12∶12 LD conditions. Flies are exposed to heat-shock (+HS) or not (−HS), and measured for in vivo luminescence. The relative luminescence is plotted as a function of time, with daytime (white bars) and nighttime (black bars) conditions indicated below the graph. Each data point represents the average of 24 flies. (TIF) [file pone.0045130.s002.tif]

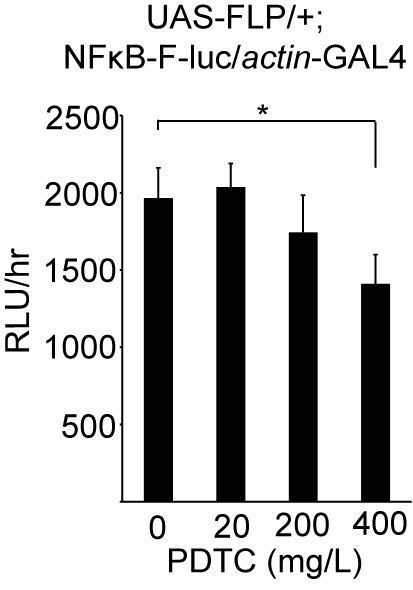

Supplement: Figure S3 — PDTC inhibition of NFκB-F-luc reporter activity persists for 24 hours at high doses. The relative luminescence is plotted with respect to the PDTC dose fed to flies. Triply transgenic flies (UAS-FLP/+;NFκB-F-luc/actin-GAL4) were fed different dosages of PDTC for 24 hours and then measured for luminescence 1 h after the end of feeding. Reporter activity is pooled over 1 day following 24 h PDTC feeding (n = 24 for each group) (Error bars = S.E.M, *p<.05). (TIF) [file pone.0045130.s003.tif]

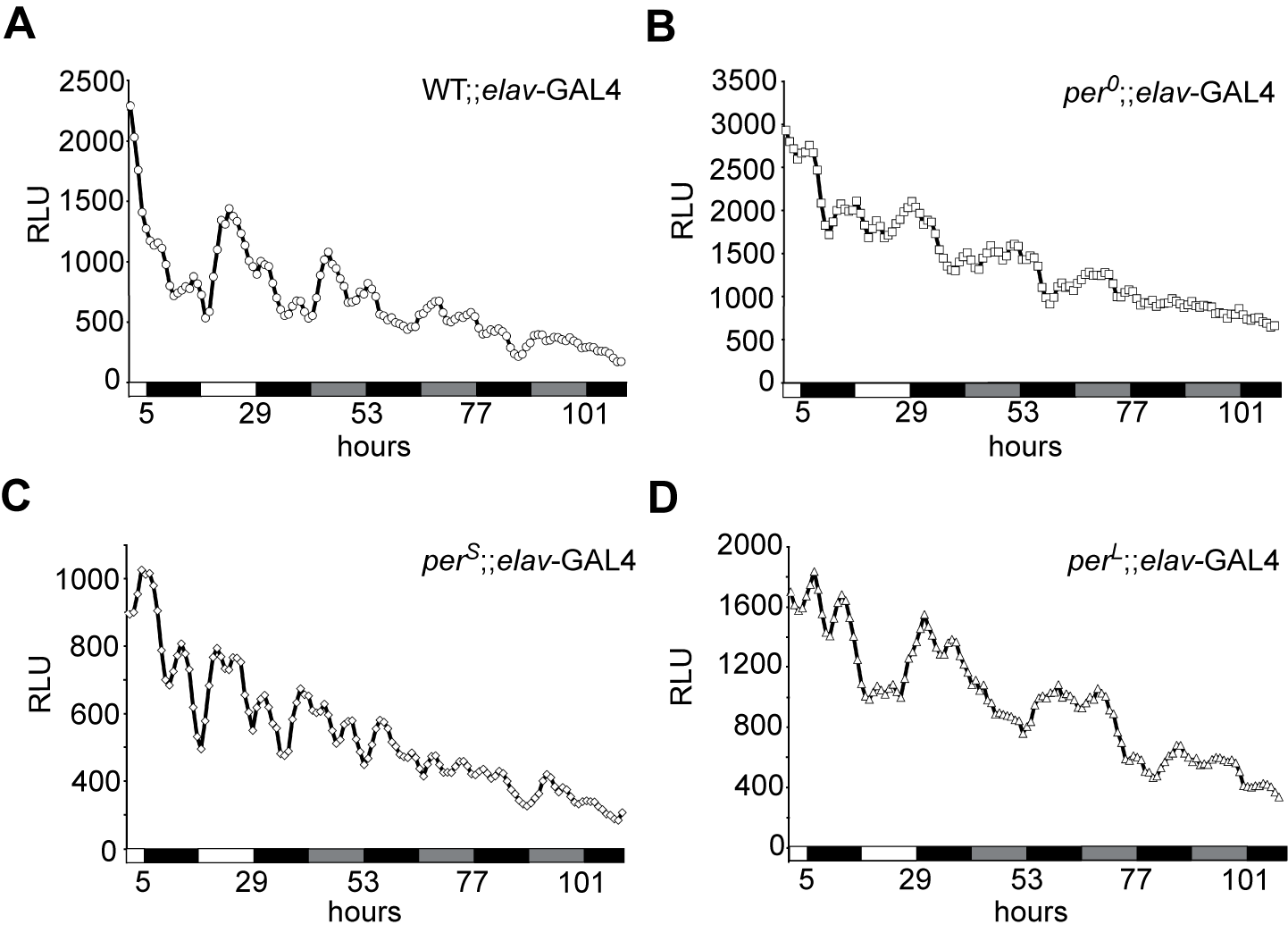

Supplement: Figure S4 — Neuronal CRE-F-luc reporter cycling is modulated in per mutants. Neuronal reporter activity is plotted over time as flies are shifted from light∶dark to constant darkness. For all of these panels, the same transgenes (UAS-FLP/+; NFκB-F-luc/elav-GAL4) exist in all flies, but the flies are examined in a wild type (A), per0 (B), perS (C) or perL (D) genetic background. (TIF) [file pone.0045130.s004.tif]

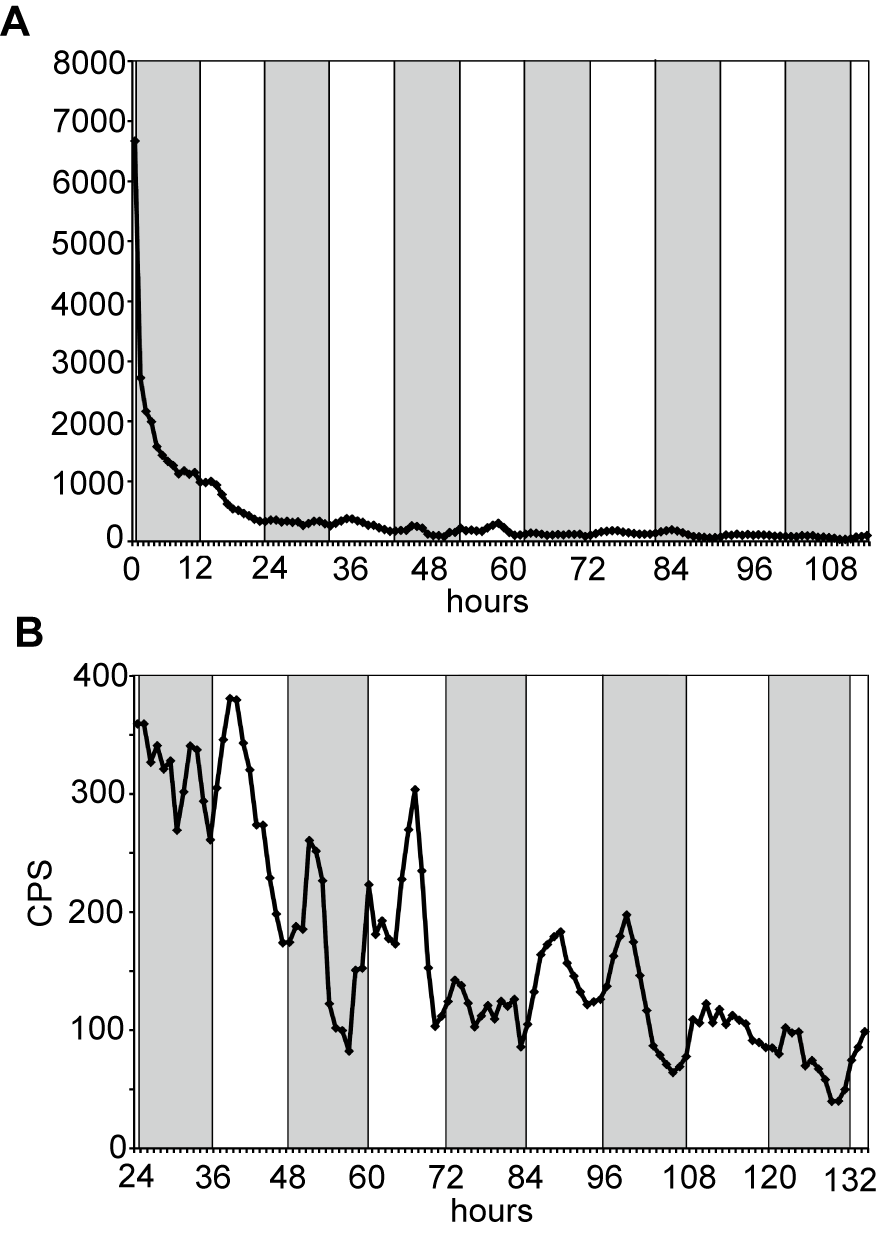

Supplement: Figure S5 — Oscillations in CRE-luc reporter activity persist after substrate removal. (A) Reporter activity (in relative light units, RLU) in CRE-luc reporter flies is plotted as a function of time, starting 1 h after the transfer from luciferin to non-luciferin food. Light conditions are indicated by white boxes (daytime) and grey boxes (nighttime). (B) The same data as in (A), with the first day excluded. (TIF) [file pone.0045130.s005.tif]
