# Supplementary material for: In Vivo Circadian Oscillation of dCREB2 and NF-κB Activity in the Drosophila Nervous System
Source: PLoS One. 2012 Oct 15;7(10):e45130. doi: 10.1371/journal.pone.0045130 (PMC3471920; doi:10.1371/journal.pone.0045130)
Supplement: Methods S1 — (DOC) [file pone.0045130.s006.doc]

**Supporting Methods**

**Eye Dissection**

Heads were removed from female flies and eyes were dissected in cold PBS. The remaining head tissue was collected and assayed separately. Luciferase activity in dissected tissue was measured using the SteadyGlo Luciferase Assay System (Promega).

**Luciferin Pre-Feeding**

CRE-luc flies were entrained to a 12:12 LD cycle for 5 days, and transferred to luciferin food (5mM luciferin, 1% agar, 5% sucrose) for 3h. After pre-feeding, flies were loaded into 96-well plates containing luciferin-free food (1% agar, 5% sucrose), and subsequent reporter activity was recorded.

**Fly lines for dCREB2 reporter activity screen**

The *c739*, *npf*-GAL4, *NinaE*-GAL4, *pdf*-GAL4, *Tdc1*-GAL4, *Tdc2*-GAL4, *gmr*-GAl4, and *repo*-GAL4 lines were obtained from the Bloomington Stock Center. The c42, c232 lines were kindly provided by V. Jayaraman. The *mz0709*-GAL4, and *alrm-*GAL4 lines were kindly provided by M. Freeman. GAL4 driver lines or wildtype lines were crossed to UAS-FLP;CRE-F-luc flies, and tested for 3-4 days under 12:12 LD conditions for *in vivo* luciferase activity. Significant signal vs. baseline was determined by comparing hourly light count between triply transgenic GAL4 driver/UAS-FLP/CRE-F-luc flies and concurrently run doubly transgenic UAS-FLP/CRE-F-luc flies. The presence of daytime (ZT=0-12) or nighttime (ZT=12-24) peaks was scored visually.
