# Supplementary material for: In Vivo Circadian Oscillation of dCREB2 and NF-κB Activity in the Drosophila Nervous System
Source: PLoS One. 2012 Oct 15;7(10):e45130. doi: 10.1371/journal.pone.0045130 (PMC3471920; doi:10.1371/journal.pone.0045130)
Supplement: Table S1 — dCREB2 Reporter Driver Screen. (DOC) [file pone.0045130.s007.doc]

Supplemental Table 1: dCREB2 Reporter Driver Screen

| Driver | Expression Pattern | Significant Signal | Reporter activity  (% background) | Daytime peak | Nightime peak |
| --- | --- | --- | --- | --- | --- |
| *hs*-GAL4 | Global | Yes | 775 | - | - |
| *actin*-GAL4 | Global | Yes | 1266 | ✓ | ✓ |
| *ok107*-GAL4 | Mushroom body: all lobes | Yes | 155 | ✓ | ✓ |
| *c739* | Mushroom body: αβ lobes | Yes | 873 | ✓ | ✓ |
| *c42* | Ellipsoid body: R2/R4m neurons | Yes | 817 | ✓ | ✓ |
| *c232* | Ellipsoid body: R3/R4d neurons | Yes | 289 | ✓ | ✓ |
| *elav*^c155^-GAL4 | Pan-neuronal | Yes | 680 | ✓ | ✓ |
| *repo*-GAL4 | Pan-glial | Yes | 1389 | ✓ | ✓ |
| *mz0709*-GAL4 | Ensheathing Glia | Yes | 1249 | ✓ | ✓ |
| *alrm*-GAL (2) | Astrocytic glia | Yes | 176 | ✓ | ✓ |
| *alrm*-GAL4 (3) | Astrocytic glia | Yes | 372 | ✓ | ✓ |
| *npf*-GAL4 | Neuropeptidergic | No | - | - | - |
| *gmr*-GAL4 | Pan-eye | Yes | 948 | ✓ | ✓ |
| *gmr^long^*-GAL4 | Pan-eye | Yes | 574 | ✓ | ✓ |
| *ninaE*-GAL4 | Pigment cells (photoreceptors) | Yes | 330 | ✓ | ✓ |

Significant signal (RLU/hr, p<0.05) for driver/UAS-FLP/CRE-F-luc flies (reporter) compared to UAS-FLP/CRE-F-luc flies (background) *in vivo* under LD conditions. Average reporter activity (RLU/hr) is expressed as a percentage of background activity. Check marks indicate the presence of an activity peak during the daytime (ZT=0-12) or nighttime (ZT=12-24).
